# A systematic review of the effects of rumination-focused cognitive behavioral therapy in reducing depressive symptoms

**DOI:** 10.3389/fpsyg.2024.1447207

**Published:** 2024-12-03

**Authors:** Yuyang Li, Chunxi Tang

**Affiliations:** ^1^College of Applied Economics, Guizhou University of Finance and Economics, Guiyang, Guizhou, China; ^2^Department of Gynaecology and Obstetrics, The First People’s Hospital of Zunyi (The Third Affiliated Hospital of Zunyi Medical University), Zunyi, Guizhou, China

**Keywords:** rumination-focused CBT, depression, rumination, prevention, treatment, relapse

## Abstract

There is still potential room for improving the effectiveness of standard Cognitive Behavioral Therapy (CBT) in preventing the onset of depression, achieving full remission, and preventing relapse or recurrence of depression. Standard CBT seems less effective in reducing depressive rumination, a key risk factor leading to the onset and persistence of depression. To improve treatment efficacy for depression, rumination-focused cognitive behavioral therapy (RFCBT) was developed, which was modified from CBT and specifically targeted to manage rumination. This systematic review aimed to assess the effects of RFCBT by evaluating whether RFCBT could contribute to reducing depressive symptoms pre-post intervention. A literature search was conducted up to April 30, 2024, across four English-language databases, including PubMed, Web of Science, Google Scholar, and Embase. The search terms employed were: (depress* OR mood OR affect OR rumination) AND (“Rumination Focused Cognitive behavio* Therapy” OR RFCBT). Among the initial 328 studies identified, 12 met the inclusion criteria, of which 10 were randomized controlled trials. Intervention characteristics and results were narratively synthesized to address the review aims. This review found preliminary evidence that the RFCBT could eliminate depressive symptoms post-intervention, and might prevent individuals from developing depression, alleviate depressive symptoms, and prevent relapse of depression, as well as reduce rumination. RFCBT could be promoted to treat depressive symptoms, especially for those with a high tendency toward rumination. However, more studies with rigorous designs are required to confirm its efficacy across different stages of depression. Future studies could compare RFCBT with other psychotherapies, dismantle the psychological therapies to identify their effective components, and explore which specific groups of people might benefit most from this intervention.

## 1 Introduction

It is estimated that around 3.8% of the global population experiences depression (World Health Organization, 2023), and the global prevalence of depression across 32 countries was approximately 28.0% (Nochaiwong et al., 2021). Depression is one of top contributor to the global disease burden (GBD 2019 Mental Disorders Collaborators, 2022), resulting in substantial personal, social, and economic consequences (Cook et al., 2019). One recommended psychological intervention for unipolar depression is Cognitive-behavioral therapy (CBT), which shows effectiveness in preventing the incidence of depression, improving remission rates, and eliminating the risk of recurrence of depression (Bockting et al., 2018; Cuijpers et al., 2016; DeRubeis et al., 2005; Moeller et al., 2019; Otto et al., 2022).

Nevertheless, there is still large room for improving the effectiveness of CBT (Langenecker et al., 2024). The effects of traditional CBT in addressing depression might be undermined by not particularly dealing with rumination, a crucial risk factor that seems strongly correlated to the development of depression (Moeller et al., 2019). Regarding preventing depression, it is suggested that the universal preventive intervention may be not as effective as the targeted and selective interventions (Horowitz and Garber, 2006). Unlike the broad-based CBT strategies, targeting modifiable risk factors can provide a more personalized method tailored to individual needs, thus facilitating the motivation and involvement to receive intervention (Vitiello, 2011). Rumination is one such risk factor that could predict the ongoing progression of major depression even after adjusting for the cognitive risk status (Abela and Hankin, 2011; Spasojević and Alloy, 2001). In terms of the remission and relapse of depression, CBT resulted in remission of depression in less than 50% of the treated people (DeRubeis et al., 2005; Cuijpers et al., 2014), and only up to 40% of people achieved lasting long-run recovery (Langenecker et al., 2024). One reason for the poor maintenance of treatment effects lies in the ineffective handling of rumination. Rumination tends to prolong and intensify existing episodes of depression (Nolen-Hoeksema, 1991; Nolen-Hoeksema et al., 2008) and is linked to a poor recovery rate and a slower treatment response to pharmaceutical and cognitive treatment (Ciesla and Roberts, 2002; Jones et al., 2008). This factor is also a common residual symptom after the remission of depression (Nolen-Hoeksema et al., 2008) and serves as a critical predictor for relapse of depression (Otto et al., 2022). Therefore, given its role in the development of depression, rumination can be targeted in both the prevention and treatment of depression (Otto et al., 2022).

Depressive rumination involves repetitively analyzing oneself, one’s issues, worries, and feelings of low mood and distress emotions (Nolen-Hoeksema, 1991; Watkins, 2008). Under the habit-goal framework of depressive rumination, depressive rumination can be framed as a mental habit (Watkins and Nolen-Hoeksema, 2014). The traditional CBT, relying on challenging the contents of thoughts, may not be necessarily effective in addressing habitual rumination. This is because CBT aimed at challenging thoughts, intentions, or beliefs or presenting new information makes it difficult to successfully alter the habitual behaviors driven by the stimulus–response mechanism (Verplanken and Wood, 2006). Rather, Wood and Neal (2007) proposed that effective approaches that can maximize the change of habit should offer individuals practical tools to manage habit cues. Aligned with this framework, the Rumination-focused Cognitive Behavioral Therapy (RFCBT), which incorporates functional analysis, stimulus control, and behavioral activation (Martell et al., 2001; Watkins et al., 2007) to directly address rumination by enabling individuals to identify cues of habitual rumination and to form new constructive thinking habits, may effectively eliminate rumination and depression (Watkins et al., 2011).

Modified from the traditional CBT to specifically deal with depressive rumination (Watkins et al., 2007), RFCBT consists of two key new adaptations: (1) conceptualizing rumination as a mental habit (Watkins and Nolen-Hoeksema, 2014), RFCBT employs functional analysis to assist individuals to recognize the cues triggering rumination and train them to respond more adaptively to those cues by using strategies such as contingency If-Then plans. This strategy can help reduce avoidance behaviors and encourage the formation of adaptive approach behaviors, by which individuals can manage rumination more effectively. (2) drawing on the empirical study that suggests the impacts of repetitive thought are determined by how the information is processed (Watkins et al., 2008), RFCBT trains individuals to adopt a more constructive processing style (Watkins, 2008). This involves transitioning from unconstructive repetitive thinking, characterized by abstract, evaluative processing that focuses on interpreting events and difficulties, to a more constructive approach involving specific, concrete, and action-oriented processing (Watkins, 2008; Watkins et al., 2008). Unlike conventional CBT, RFCBT does not heavily rely on challenging negative thoughts directly; instead, it emphasizes transforming the thinking process rather than solely addressing the content (Hvenegaard et al., 2019).

As an intervention that is directly modified to tackle rumination, RFCBT may deserve a deeper understanding to inform future research and practical implementation. Although rumination is regarded as a transdiagnostic mechanism that may lead to the development and recurrence of various mental illness, including depression, social anxiety, generalized anxiety disorder, and post-traumatic stress disorder (McLaughlin and Nolen-Hoeksema, 2010; Watkins, 2015), the focus on depression is driven by evidence indicating that rumination may be more closely related to depressive disorders (Nolen-Hoeksema et al., 2008; Olatunji et al., 2013), and rumination seems a specific predictor for future changes in depressive symptoms while not for anxious arousal or externalizing problems (Hankin, 2008). Given the global burden of depression, which surpasses many other mental disorders (GBD 2019 Mental Disorders Collaborators, 2022), and the higher relapse rates observed in depression compared to anxiety disorders after traditional CBT treatment—33% versus 14% (Levy et al., 2021; Wojnarowski et al., 2019), there is a compelling case for the specific application and attention of RFCBT in treating depression.

To our knowledge, there seemed no systematic review that specifically targeted the RFCBT on depressive symptoms. Hence, the primary aim of this study was to provide an overview to assess the impacts of RFCBT by evaluating whether RFCBT could decrease depressive symptoms pre-post interventions. The treatment effects of RFCBT would be discussed across three stages - prevention from the onset of depression, remission of depression, and prevention from relapse or recurrence of depression - to align with recent recommendations for tailoring CBT to provide stage-specific treatment strategies to manage depression (Otto et al., 2022). This study also discussed the key underlying psychological and neurobiological factors in RFCBT that contributed to addressing depression.

## 2 Method

To address the research questions, this paper conducted a systematic review, which employed a structured search strategy to gather all significant published papers related to RFCBT and depressive symptoms, and also incorporated a narrative synthesis of the research findings, based on the guidelines of Preferred Reporting Items for Systematic Reviews and Meta-Analysis (PRISMA) (Page et al., 2021).

### 2.1 Search strategies

Comprehensive searches were conducted across several databases up to April 30, 2024, including PubMed, Web of Science, Google Scholar, and Embase based on the PICO-S framework (Lefebvre et al., 2023) (i.e., Population, Intervention, Comparisons/control, Outcome, Study design) to structure the search process. The population defined in this study was people exhibiting depressive symptoms, those diagnosed with depression, or those at high risk for depression. The intervention was rumination-focused cognitive–behavioral therapy (RFCBT). Comparisons were any type of comparator or no comparator at all. The outcomes were depressive symptoms and levels of rumination. The study design criteria excluded those published as abstracts, study protocols or conference papers in which RFCBT had not been implemented, or review papers.

To refine the search, the following terms were utilized: (depress* OR mood OR affect OR rumination) AND (“Rumination Focused Cognitive behavio* Therapy” OR RFCBT). The search syntax was adapted as necessary for each specific database to ensure comprehensive and relevant academic papers were identified and retrieved.

### 2.2 Inclusion and exclusion criteria

Inclusion criteria included: (1) Research must be published in English; (2) Research primarily targeted to the RFCBT; (3) Research should use validated measures to assess depressive symptoms. Exclusion criteria were: (1) The intervention used in the research integrated elements of other therapies not specifically in RFCBT; (2) Research was not centrally concerned with depressive symptoms, unipolar depression, or the depressed group; (3) Research was study protocols and conference papers in which the planned RFCBT had not yet been implemented, reviews of related topics, and unpublished manuscripts without peer review.

### 2.3 Study selection

**Figure 1:**
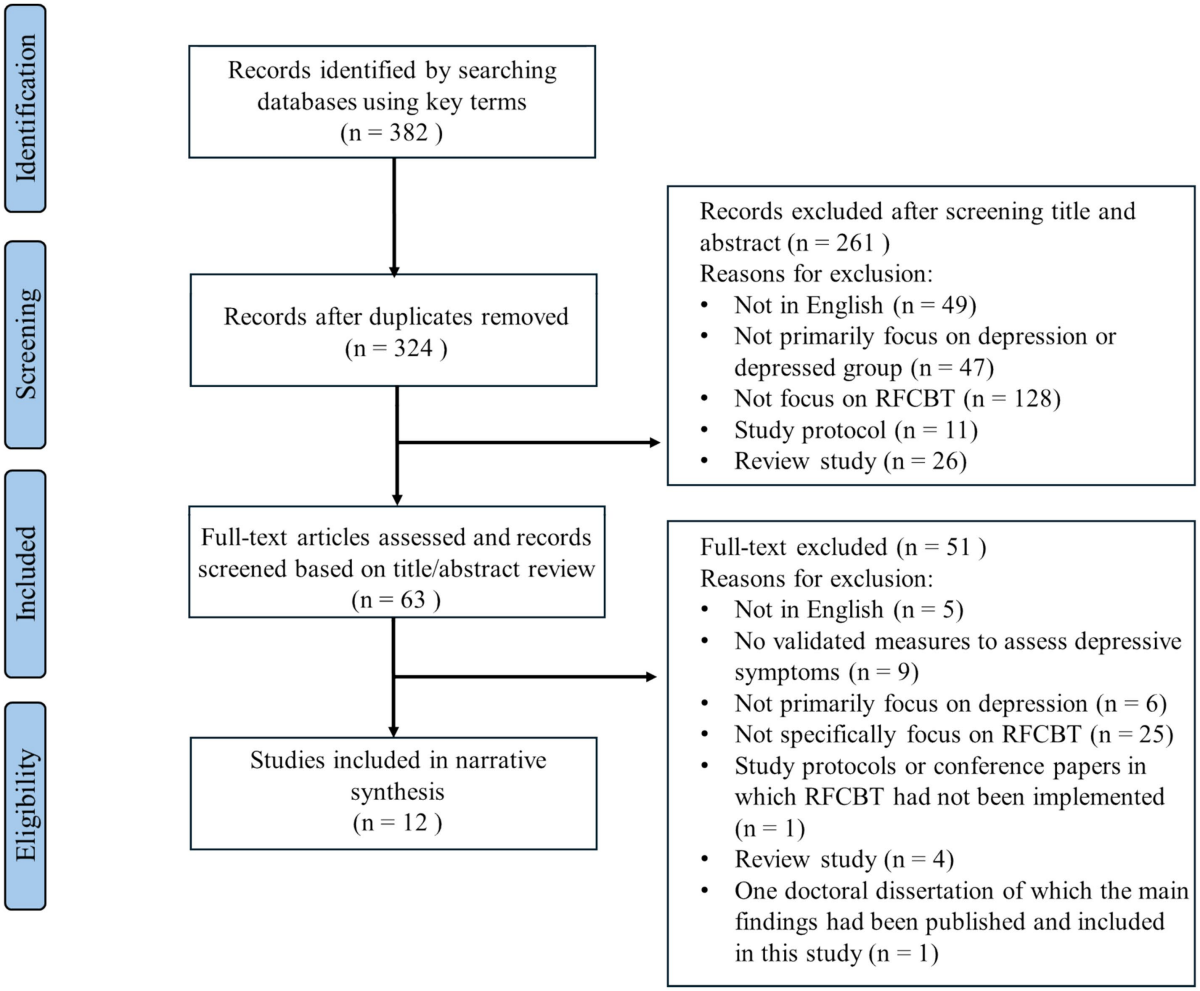
The flowchart diagram.

Initial records from the four databases were compiled into an Excel file, with a total of 382 articles. After removing duplicates, 324 articles remained. Then two researchers independently reviewed titles, abstracts, and the full text of studies from the four databases to assess the eligibility of these papers according to the selection criteria. Researchers reached a consensus on which articles should be included and the controversial papers were consulted with another independent investigator. The reasons for exclusion and screening outcomes were recorded in Excel by each researcher. The screen of titles and abstracts excluded 261 studies, leaving 63 articles for full-text review. Subsequent evaluations of full texts excluded an additional 51 records, resulting in 12 articles meeting all inclusion criteria. Among the confirmed articles, references were manually checked to ensure that no relevant articles were missed. The entire selection process, including the systematic elimination of irrelevant studies, is depicted in Figure 1.

### 2.4 Data extraction and synthesis

The researcher LY and TC individually extracted the key data from the included studies, which consisted of (1) authors and year of publication; (2) the country; (3) the sample size; (4) sample characteristics (gender & age); (5) the study design; (6) formats of intervention; (7) duration of interventions; (8) follow-up(s); (9) targeted stages in addressing depression; (10) measurements; (11) main findings. Any discrepancies encountered were discussed and consulted to reach a consensus, as detailed in Table 1. The collected data were then synthesized to create a comprehensive narrative summary of RFCBT interventions about their effects in alleviating depressive symptoms at various stages of depression development.

**Table 1:** Summary of demographics and characteristics of the studies.

Study (Author, year)	Country	Sample size	Gender (F/M)	Age (RFCBT/Control)	Study design	Forms of RFCBT	Duration of RFCBT	Follow-up	Targeted stage	Measurements of depression and rumination	Main findings
Watkins et al. (2011)	UK	42	24/18	43.05 /45.24	Treatment as usual v. Treatment as usual plus RFCBT	In-person	12 sessions	-	Treating residual depression	HRSD; BDI-II; The Structured Clinical Interview for DSM-IV; RRS	Remission rates and residual symptoms were significantly improved when adding RFCBT to TAU compared to TAU only. The changes in rumination mediated the treatment effects.
Umegaki et al. (2022)	Japan	39	39	18.38	Uncontrolled	Self -help	3 modules, with 1–20 weeks to complete each module	2, 4, and 6 months	Prevention the onset of depression	RRS; PHQ-9	Rumination had significantly decreased post-intervention, while there was no significant decrease in depression post-intervention.
Mak et al. (2023)	China (Hong Kong)	256	186/70	28.33/26.28 /28.21	RFCBT v. Mindfulness-based intervention v. Psychoeducation	Internet-based	6 weeks	3-month & 9-month follow-ups	Prevention the onset of depression	The Chinese version of PHQ-9; RRS	The three conditions showed similar improvements in depression. While rumination changed differently between the RFCBT and MBI group, Participants in RFCBT demonstrated a significant decrease in rumination after 3 month, while there was no significant long-term effect at a nine-month follow-up among participants in MBI.
Cook et al. (2019)	UK	235	196/39	20.43 /20.53 /20.27	Guided internet-based RFCBT v. unguided internet-based RFCBT v. usual control	Internet-based	Around 3 months	6-month & 15-month follow-ups	Prevention the onset of depression	RRS; PHQ-9	The risk of depression was reduced by 34% in the guided i-RFCBT group. Rumination and depressive symptoms showed significant improvements in the short-to-middle run. Unguided i-RFCBT showed comparable effect sizes with guided i-RFCBT.
Hvenegaard et al. (2019)	Denmark	131	100/31	39.8 /39.4	Group RFCBT v. group CBT	Group	12 sessions	6-month follow-up	Treating depression	HRSD; HAM-D6; RRS	There was a larger improvement in observed-rated depressive symptoms in the RFCBT group than the group CBT control post-intervention. At six-month follow-up, both two groups showed no treatment differences in rumination or depressive symptoms.
Moeller et al. (2019)	Denmark	10	Not disclosed	45.5	Uncontrolled	In-person	12-16 sessions over a period of 4 months	3-month follow-up	Treating non-responsive chronic depression	HRSD; HAM-D6; RRS	Depressive symptoms and rumination were significantly declined post-intervention and at follow-up.
Bessette et al. (2020)	US	33; 29 completing post-intervention fMRI scan; 23 completing 2-year follow-up fMRI scan	19/14	15.27 /15.93	RFCBT v. Assessment Only	In-person	8 weeks	2-year follow-up	Prevention of relapse	CDRS-R; RADS; RRS	Youths with RFCBT maintained lower depressive symptoms, slower relapse rates, and less frequent rates than AO over 2 years. The reduction was maintained in RFCBT at a 2-year follow-up, while ruminative tendencies were returned in AO. Adolescents with increased activity in limbic areas and default mode at baseline in rumination induction task showed fewer depressive symptoms over the follow-up.
Langenecker et al. (2024)	US	76	51/25	15.67 /16.00	RFCBT v. Treatment as usual	In-person plus telehealth	10 to 14 sessions	-	After remission	RRS; CDRS-R	Participants in the RFCBT group showed significantly reduced rumination and a decreased connectivity between PCC and right IFG/ITG compared to those in the TAU group.
Jacobs et al. (2016)	US	33; 22 completing the fMRI scans pre- and post-intervention	19/14	15.41 /15.69	RFCBT v. Assessment Only	In-person	8 weeks	-	Prevention of relapse	RADS; CDRS-R; RRS	A reduction in self-reported depression and rumination was reported in adolescents under RFCBT over 8 weeks, who also exhibited a significant reduction in connectivity between left PCC and the right IFG and bilateral ITG.
Topper et al. (2017)	Holland	251	210/41	17.32 /17.36 /17.67	Group RFCBT v. internet-based RFCBT v. waitlist control	Group or Internet-based	6 weeks	3-month follow-up & 12-month follow-up	Prevention the onset of depression	RRS; PTQ; BDI-II; MASQ-D30; PHQ-9	Rumination, depressive symptoms, and incidence rate of depression were significantly reduced in both group RFCBT and i-RFCBT at post-intervention, and these effects were maintained at 12-month follow-up. No significant difference in outcome measures was found between these two treatment conditions. Mediation analyses showed that repetitive negative thinking mediated the intervention effects on the incidence of major depression.
Watkins et al. (2007)	UK	14	9/5	42.86	Uncontrolled	In-person	12 weekly 60-min sessions	-	Treatment for residual depression	The Structured Clinical Interview for DSM-IV; HRSD; BDI-II; RRS	The RFCBT significantly improved depressive symptoms, rumination, and co-morbid disorders.
Jung (2019)	China (Hong Kong)	139	102/37	27.92 / 28.82	i-RFCBT v. i-psychoeducation	Internet-based	6 weeks	3-month follow-up & 9-month follow-up	Prevention the onset of depression	PHQ-9; RRS	Both i-RFCBT and i-psychoeducation could reduce the rumination and depressive symptoms of high-risk participants post-intervention, while no significant difference in changes in the two scores was found between groups. Behavioral activation was significantly increased under the i-RFCBT group. The rumination could fully mediate the effect of i-RFCBT on depressive symptoms.

HRSD, Hamilton Rating Scale for Depression; BDI-II, Beck Depression Inventory; RRS, Ruminative Response Scale of the Response Style Questionnaire; PHQ-9, Patient Health Questionnaire; HAM-D6, Hamilton Self-report Questionnaire; CDRS-R, The 17-item Children’s Depression Rating Scale–Revised; RADS, the 30-item Reynolds Adolescent Depression Scale; PTQ, Perseverative Thinking Questionnaire; MASQ-D30, Mood and Anxiety Symptom Questionnaire-D30.

## 3 Results

### 3.1 The effects of RFCBT in addressing depression at different stages

This review consisted of 12 studies, with 10 of these studies adopting randomized controlled trials (see Table 1). 5 studies assessed whether RFCBT could reduce depressive symptoms to prevent depression onset, with 4 articles reporting a significant reduction in depressive symptoms post-intervention (Mak et al., 2023; Cook et al., 2019; Topper et al., 2017; Jung, 2019). Mak et al. (2023) revealed that participants’ depression scores significantly decreased over time, noted at the end of the program and during follow-ups at 3 and 9 months. Similarly, Topper et al. (2017) also found that the decrease in depressive symptoms and the reduced incidence of depression under RFCBT condition could be maintained at 12-month follow-up. Instead of a long-term effect, Cook et al. (2019) showed that a significant improvement of depressive symptoms under the guided internet-based RFCBT (i-RFCBT) compared to usual care could be only found at 3 and 6 months. In contrast, Umegaki et al. (2022) did not find a significant decrease in depression symptoms post-intervention.

Regarding the resolution of depression, 71% of participants reached the standards for therapeutic response, and 50% achieved complete remission in Moeller et al.‘s (2019) study. Additionally, the initial treatment effects on depressive symptoms were sustained at the three-month follow-up (Moeller et al., 2019), and at the six-month follow-up (Hvenegaard et al., 2019). When treating the residual depressive symptoms, Watkins et al. (2011) showed that the intervention group that combined RFCBT with treatment-as-usual (TAU) notably enhanced residual symptoms and remission rates relative to the TAU group alone.

In regard to preventing the relapse or recurrence of depression after remission, the studies of Bessette et al. (2020) and Jacobs et al. (2016), which mainly targeted adolescents and youths, demonstrated significant benefits of RFCBT compared to the assessment-only (AO) group. Participants in the RFCBT group reported lower depressive scores over 8 weeks (Jacobs et al., 2016). This improvement could be also maintained in a two-year follow-up compared to the AO group, and the youth treated by RFCBT also experienced relapses more slowly and less frequently and had fewer instances of hospitalization due to suicidality (Bessette et al., 2020). Similarly, Langenecker et al. (2024) also found that youths who had remitted from depression and were treated with RFCBT had a greater decrease in rumination scores than those in the TAU group.

### 3.2 The comparisons of the effects between different delivery formats of RFCBT

Cook et al.’s (2019) study compared the efficacy of guided web-based RFCBT and unguided web-based RFCBT. The former group was supported by qualified clinicians specifically trained in RFCBT and supervised by the developer of RFCBT, and the latter group only contained automatic web-based conditional feedback to answer common exercise questions, along with necessary weekly assessments for suicidal ideation. The study results showed that unguided web-based RFCBT yielded similar favorable effect sizes on improving rumination and depressive symptoms as guided web-based RFCBT. Specifically, guided web-based RFCBT and unguided web-based RFCBT decreased the risk of depression by 34% (HR = 0.66, 95%CI [0.35, 1.25]) and 36% (HR = 0.64, 95%CI [0.33, 1.24]) compared to usual care, respectively.

Likewise, no disparities in outcome measures were found between the group-based RFCBT and internet-based RFCBT (Topper et al., 2017). Both formats led to a largely lower incidence rate of depression over 12 months (group-based: 15.3%, internet-based: 14.7%), in contrast to the waitlist control (32.4%). Both intervention groups also caused a much greater reduction in repetitive negative thoughts than the control, without notable differences found between these two active interventions.

### 3.3 The comparisons of the effects of RFCBT with CBT, MBI and psychoeducation

In addition to comparing different formats of RFCBT, research also assessed the efficacy of RFCBT in comparison to other psychological therapies, including CBT, MBI and psychoeducation (Hvenegaard et al., 2019; Jung, 2019; Mak et al., 2023). Hvenegaard et al. (2019) discovered that clinically diagnosed participants receiving RFCBT showed a significantly larger decrease in observer-rated depressive symptoms post-intervention than those receiving group CBT, after controlling for baseline disparity in observer-rated depressive symptoms scores. Nevertheless, both groups showed reductions in rumination and self-reported depression and there was no statistical difference between RFCBT and CBT in these two variables after treatment (Hvenegaard et al., 2019). Furthermore, no significant differences in average depressive symptoms were evident between the RFCBT group and the CBT group in the intention-to-treat (ITT) sample at the six-month follow-up (Hvenegaard et al., 2019).

Regarding prevention from depression, Jung (2019) found that although both the i-RFCBT and i-psychoeducation groups could lower the level of depressive symptoms and rumination of high-risk individuals after the intervention, there was no significant interaction effect between time and groups (i-RFCBT vs. i-psychoeducation) on depression and rumination. Similarly, Mak et al. (2023) revealed no significant time x condition interaction in decreasing depressive symptoms among the RFCBT, Mindfulness-Based Intervention (MBI), and psychoeducation groups. However, a significant time x group interaction was observed solely for rumination when comparing the RFCBT group and MBI group. Particularly, the RFCBT group showed a decline in rumination pre-post intervention similar to the other two groups. Nevertheless, a rebound of rumination scores was observed in the RFCBT group at the three-month follow-up with the rumination scores decreasing again by the nine-month follow-up. For the MBI group, rumination scores rose back to the baseline levels at the nine-month follow-up, suggesting that patterns of changes in rumination differed among the three treatment groups, and the decrease in rumination was not sustained in MBI group at the nine-month follow-up (Mak et al., 2023).

### 3.4 The changes in psychological and neurobiological indicators under RFCBT

In addition to diminishing rumination scores (Hvenegaard et al., 2019; Langenecker et al., 2024), RFCBT was found to eliminate depressive symptoms through the mediation of rumination (Watkins et al., 2011; Jung, 2019; Topper et al., 2017). Watkins et al. (2011) and Jung (2019) highlighted that the changes in depressive rumination could mediate the treatment effects of RFCBT on depressive symptoms. When depressive rumination was added as a variable, the treatment condition ceased to be a significant factor in predicting changes in depressive symptoms. Likewise, Topper et al. (2017) also revealed that repetitive negative thinking played a mediating role in the impacts of RFCBT on the occurrence of major depression and could account for 38.9% of the effect on the incidence of depression. Furthermore, one study also revealed that the i-RFCBT group showed a significant increase in behavioral activation across time (*F*(2.00, 104.19) = 15.40, *p* < 0.001), however, the behavioral activation did not mediate the effects of i-RFCBT on changes in depression scores (Jung, 2019).

Functional magnetic resonance imaging (fMRI) was used to assess the underlying neural responses (Bessette et al., 2020; Langenecker et al., 2024; Jacobs et al., 2016). Bessette et al. (2020) found that high correlations existed between pDMN+ (posterior default mode, limbic, and visual areas) and SV-SM (salience-emotion, visual and somatomotor regions) networks at both baseline and week 8 (*r* = 0.95) among youth remitted depression. Each network’s activation did not significantly relate with itself across time, indicating individual differences in response to intervention. However, Bessette et al.‘s (2020) study results failed to support that remitted participants with the greatest change in rumination significantly reduced the differences in pDMN + activation compared to the healthy control activation. Instead, higher baseline activation in pDMN+ under the Rumination-Distraction task was linked to lower depression scores at 8 weeks, 1 year, and 2 years (*B* = −2.15), in which the RFCBT group demonstrated lower depressive levels than the AO group.

Jacobs et al. (2016) observed that remitted adolescents who had MDD under the RFCBT group exhibited decreased connectivity between the left Posterior Cingulate Cortex (PCC) to other Default Mode Network (DMN) areas including middle cingulate, orbitofrontal cortex (OFC), and areas in the cognitive control network (CCN) such as the right inferior frontal gyrus (IFG), and bilateral inferior temporal gyri (ITG). Moreover, the weakened connectivity between the left PCC and the right ITG was associated with reductions in rumination (*r* = 0.48) and self-rated depressive symptoms (*r* = 0.69). Langenecker et al. (2024) replicated the prior results, showing a notable decline in rumination scores and reduced connection between the left PCC and the right IFG/ITG in participants undergoing RFCBT compared to those receiving TAU. However, exploratory analyses revealed that no significant correlation between the reduction in rumination and reduction in connectivity (*r* = −0.21).

## 4 Discussion

This review aimed to analyze the effects of the RFCBT in addressing depressive symptoms at the different stages of depression development, including the prevention of the onset of depression, treatment of depression syndromes and residual depression symptoms, and prevention of relapse of the depression. There were 12 studies eligible for inclusion in this review. Overall, studies indicated that RFCBT could contribute to eliminating depressive symptoms and treating depression.

### 4.1 The treatment effects of RFCBT

Study results might provide preliminary evidence that RFCBT might be effective in preventing the onset of depression in high-risk populations and showed medium-to-long-term effects (Mak et al., 2023; Cook et al., 2019; Topper et al., 2017), although one study failed to support these promising effects (Umegaki et al., 2022). One possible explanation for the insignificant decline in depressive symptoms might be that the baseline scores of depressive symptoms had already been relatively low (Umegaki et al., 2022). This suggests that RFCBT could be especially beneficial for individuals at high risk of developing depression, particularly for those with a high tendency of rumination, and therefore RFCBT may serve as an effective intervention for this specific group. Additionally, RFCBT may also play a role in reducing depressive symptoms during depressive episodes (Hvenegaard et al., 2019). Particularly, Moeller et al. (2019) suggested that RFCBT worked for individuals with non-responsive chronic depression. This may support the hypothesis that targeting rumination, a factor not typically emphasized in traditional CBT but crucial in exacerbating current episodes, may offer an effective approach to managing difficult-to-treat depression. Furthermore, by targeting depressive rumination, RFCBT might treat residual depressive symptoms (Watkins et al., 2007; Watkins et al., 2011), and RFCBT seemed able to decrease the likelihood of future relapse of the depression (Watkins et al., 2011; Bessette et al., 2020; Jacobs et al., 2016). Nevertheless, due to the small number of studies included in this review, the above interpretations should be cautious, and more studies are required to substantiate these findings. Moreover, the current studies might lack mid-to-long-term follow-ups to adequately track treatment effects. To gain robust evidence for RFCBT’s effects in preventing the relapse of depression, long-term follow-up data might be needed.

Additionally, it seemed that the intervention formats of RFCBT would not largely affect its intervention effectiveness based on two studies (Cook et al., 2019; Topper et al., 2017). The guided RFCBT and unguided RFCBT, as well as the group RFCBT and internet-based RFCBT, showed similar treatment effects. The favorable treatment effects of unguided RFCBT and internet-based RFCBT provide initial evidence that RFCBT has the potential to work as a convenient, accessible, and cost-effective tool to prevent the onset or relapse of depression. There was also an absence of conclusive evidence to confirm the efficacy of RFCBT compared to other psychotherapies. Only one study directly compared RFCBT with CBT in a group intervention, and the results did not demonstrate significant superiority of RFCBT over standard CBT in reducing self-reported depressive symptoms, even though RFCBT was found more effective in improving the observer-rated depressive symptoms. The lack of significant difference between group RFCBT and group CBT in reducing self-reported depression immediately post-treatment or at the six-month follow-up might be attributed to a considerable portion (47%) of participants being lost to follow-up, which underpowered the study to detect any real differences in self-reported depressive symptoms between the groups (Hvenegaard et al., 2019). It is also possible that there are equal effectiveness of both CBT and RFCBT in treating depression in the long term, with RFCBT potentially showing earlier benefits (Hvenegaard et al., 2019). However, the current study failed to allow for definitively distinguishing between these interpretations.

It should be noted that only Hvenegaard et al. (2019) compared the effects of RFCBT with active controls in clinically diagnosed individuals. In contrast, the insignificant outcomes between RFCBT, MBIs, and psychoeducation reported by Jung (2019) and Mak et al. (2023) were based on individuals at high risk but not diagnosed with depression. It remains unknown whether RFCBT outperforms MBIs or other psychological interventions in treating clinically diagnosed populations, especially those with treatment-resistant depression. The treatment efficacy of RFCBT at different depression progression stages might be different, considering the potential superiority of RFCBT in producing long-term effects on rumination (Mak et al., 2023). In conclusion, this review provides only preliminary insights into the comparative effects of RFCBT and alternative psychotherapies such as MBIs and standard CBT. More robust RCTs are required to determine whether RFCBT is superior to other psychological therapies in treating depression.

### 4.2 Psychological factors underlying the RFCBT

Studies have supported that RFCBT could improve the rumination tendency at the post-treatment (Watkins et al., 2011; Hvenegaard et al., 2019; Langenecker et al., 2024; Topper et al., 2017). Specifically, three studies have demonstrated the mediating role of rumination in treatment outcomes (Watkins et al., 2011; Jung, 2019; Topper et al., 2017), implying depressive rumination might be a critical factor impacting the efficacy of RFCBT. According to the habit-goal framework (Watkins and Nolen-Hoeksema, 2014), goal discrepancies could lead to rumination, as individuals might ruminate to analyze the reasons behind unmet goals and gather information to better achieve these goals. The unachieved goals may often be associated with low mood, and when ruminative thoughts repeatedly occur in the context of negative mood triggered by unresolved goals, rumination may develop into a habitual response. This goal-habit framework suggests that a negative mood state could automatically trigger the ruminative thoughts, and once established, this habit of rumination could continue, regardless of whether the initial unresolved goals that triggered these negative repetitive thoughts have been resolved or given up. This framework supports why RFCBT focuses on breaking ruminative thoughts in a way to change habits.

The treatment implications for depressive rumination suggest that a long-run decrease in depressive rumination is likely to happen if contextual triggers can be permanently eliminated or continuously avoided. Whereas the suggestion of this method requires individuals to accurately recognize the antecedent cues for rumination (Watkins and Nolen-Hoeksema, 2014). Hence, instead of directly challenging the content of negative thoughts, RFCBT relies on functional analysis to assist individuals in identifying the cues and triggers of the rumination, managing exposures to the cues, and engaging repeatedly in alternative behaviors (Hvenegaard et al., 2019), which contribute to breaking the habitual rumination. Furthermore, to effectively alter the context–response link and hence decrease habitual rumination, it is essential to replace the maladaptive ruminative response with more constructive habits and adaptive new habits can be formed by teaching individuals flexible coping strategies (Watkins and Nolen-Hoeksema, 2014). RFCBT incorporates experiential and imagery exercises, as well as behavioral experiments, to foster concrete thinking and to train people to adopt a more adaptive cognitive style and enhance their compassion for themselves or others.

According to Watkins and Nolen-Hoeksema (2014), improving negative moods, whether through antidepressants, psychological interventions, distraction, or environmental change, might at least temporarily lessen the occurrence of the habitual rumination by removing the mood contextual cues. Thus, many interventions could relieve the depressive symptoms in the short run. However, these interventions might leave individuals susceptible to relapse since they fail to directly address the depressogenic habits such as depressive rumination, which may lead to the development of more severe depressive symptoms and significant depressive episodes (Nolen-Hoeksema et al., 2008). In contrast, focusing on rumination might enable the RFCBT to have an efficacy in preventing depression among at-high-risk populations, in handling difficult-to-treat depression (Watkins et al., 2011; Watkins et al., 2007; Mak et al., 2023; Cook et al., 2019; Moeller et al., 2019; Topper et al., 2017) and in reducing the risk of depression relapse (Watkins et al., 2011; Bessette et al., 2020; Jacobs et al., 2016).

RFCBT is distinguished from CBT by its special adaption to treat rumination, however, Hvenegaard et al. (2019) indicated that this intervention did not lead to a more significant reduction in self-reported rumination than the group CBT. As previously stated, one possible explanation for the limited impact on rumination may be that the study might lack sufficient power to identify a true difference in rumination because of missing data and attrition during follow-up. Furthermore, it is plausible that group CBT could also effectively reduce rumination by challenging negative thoughts, improving problem-solving skills, and implementing activity schedules, which collectively may disrupt the cycle of rumination (Hvenegaard et al., 2019). The absence of a significant difference in treatment effects on rumination between RFCBT and CBT also raises the possibility that the effectiveness of RFCBT may not lie solely in its capacity to reduce rumination. Given that, only the overall effects of the whole intervention package were assessed and only a small number of studies were based, it is challenging to pinpoint which components of RFCBT are crucial for its effectiveness compared with psychotherapies such as MBIs and CBT. Therefore, further process-outcome research dedicated to exploring these mechanisms of change is needed to determine the specific elements within RFCBT that contribute to treatment outcomes.

### 4.3 Neurobiological factors underlying the RFCBT

Research indicated that rumination could be modified in the neuropathophysiology of major depression (Jacobs et al., 2016). RFCBT has been built on theoretical frameworks that rumination can be seen as a mental habit and has been designed to offer a comprehensive treatment program that can effectively assist individuals in identifying rumination and cultivating healthier mental habits (Jacobs et al., 2016). Neurobiological studies have demonstrated that RFCBT elicited specific alterations in brain networks associated with rumination, particularly the DMN and the CCN.

#### 4.3.1 Changes in the DMN

The DMN, incorporating key areas including the part of the ventromedial prefrontal cortex (vmPFC) and PCC, is known for its role in passive waiting, self-referential processing, and awareness of the external surroundings (Koban et al., 2021; Langenecker et al., 2024). Research indicated that rumination was correlated with a range of areas within the DMN at rest and during rumination induction tasks (Jacobs et al., 2016), and one recent work also suggested normalizing DMN hyperconnectivity contributed to alleviating depressive symptoms after repetitive transcranial magnetic stimulation (Liston et al., 2014). Studies indicated that RFCBT might modify the activation and connectivity patterns within the DMN. Although Bessette et al.‘s (2020) results failed to support their primary hypothesis that RFCBT can work as a disease-modifying treatment, this study found that the heightened baseline DMN activation during the rumination-distraction task was associated with reduced depressive symptoms over 2 years, and these effects were almost doubled in youth under RFCBT. This suggested this increased activation at baseline served as a disease marker (Burkhouse et al., 2016). The neural discrepancies observed between the remitted youth and healthy controls at baseline may represent a form of compensation to maintain health, and RFCBT may act as a treatment to sustain or bolster this compensation, which means stabilizing the activation of pDMN+.

Additionally, since rumination can be recognized as a mental habit, the way RFCBT manages rumination is a process of transforming maladaptive habits into adaptive forms. This transformation can be seen as a non-reinforced preference change, which involves changing behaviors or preferences without external reinforcements but depending on internal adjustments such as altering the cue-response (Schonberg and Katz, 2020). Therefore, the possible compensation effects of RFCBT demonstrated by heightened DMN activation might align with the previous findings that PCC activities play a vital role in the non-reinforced learning process (Zahedi et al., 2023), which is a crucial area that may facilitate internally directed cognition (Leech and Sharp, 2013). To sum up, RFCBT might enhance resilience and reduce relapse risk by better managing neural processes underlying rumination (Bessette et al., 2020).

#### 4.3.2 Changes in connection between the DMN and the CCN

The CCN is another unique neural network that widely supports comprehensive executive functions including inhibitory control, working memory, and sustained attention (Langenecker et al., 2024). RFCBT has been shown to reduce the connectivity between the DMN and key areas of the CCN including the IFG in remitted adolescents with major depressive disorder, suggesting a more independent functioning of these networks (Jacobs et al., 2016). The IFG has been linked to healthy emotional regulation (Grecucci et al., 2013). Recent research indicates that this region may play a crucial role in differentiating neural patterns between youth remitted from major depression and healthy controls (Belden et al., 2015). In addition, in adults with major depressive disorders, the increased connectivity in the DMN and the increasing dominance of DMN relative to the task-positive network were linked to increased depressive rumination (Hamilton et al., 2011; Zhu et al., 2012). Therefore, Jacobs et al.‘s (2016) finding of reduced connectivity between PCC and IFG after RFCBT raised the possibility that RFCBT might contribute to re-establishing a more adaptive pattern between DMN and CCN, or RFCBT might enable individuals to manage ruminative thoughts more easily compared to strategies that need effortful control.

Similar to Jacobs et al. (2016), RFCBT were found to not only clinically improve rumination but also lead to a significant decrease in network interaction between the several anterior and lateral CCN nodes and the posterior DMN (Langenecker et al., 2024). Langenecker et al. (2024) further reflected this reduction could be explained by a compensation framework or a decrease in looping within neural networks (Langenecker et al., 2024). According to the compensation framework, the increased connectivity between CCN and DMN happens in real time to prevent adolescents from cognitive decline. The observed decrease in connectivity following treatment could reflect enhanced coherence within these networks, reducing the necessity for such compensatory mechanisms. Another explanation may be that the diminished crosstalk between networks associated with rumination may imply that RFCBT could contribute to reducing the repetitive neural signaling that typically integrates self-reflective and regulatory processes. This could facilitate task-focused processing, shifting from habitual rumination patterns. However, the current data were unable to clearly distinguish between the above processes.

Furthermore, a reduction in connectivity from the PCC to the right ITG was associated with declines in both rumination and depressive symptoms (Jacobs et al., 2016). A previous study based on resting-state fMRI also found a significantly altered functioning in both the PCC and ITG of adolescents with depression (Gong et al., 2014). Another study identified increased connectivity between ITG and the amygdala in medication-naive adolescents who experienced first-episode depression compared to the healthy controls (Jin et al., 2011). Overall, these studies may indicate that ITG may play a role in early-onset depression and the changed connectivity between PCC and right ITG after receiving RFCBT may reveal the potential neurobiological basis for this intervention to treat depression.

Overall, the neurobiological mechanisms by which RFCBT affects the brain suggest that it does more than alleviate symptoms; it potentially reconfigures the underlying neural architecture associated with rumination. By diminishing DMN hyperconnectivity and improving DMN-CCN dynamics, RFCBT fosters a neuro-environment conducive to sustained remission and improved regulatory capacities. Future research should aim to further elucidate these mechanisms, exploring the durability of these changes and their implications for preventing depressive relapse.

## 5 Limitations and future directions

Although current evidence could preliminarily support the effects of RFCBT in alleviating depressive symptoms, this review could not conclusively affirm the effects of RFCBT due to the limited number of papers available. More studies with rigorous designs and focusing on different stages of depression development are required to strengthen the evidence for RFCBT. In addition to the small sample size, there are other limitations in the current evidence that warrant further concerns. Firstly, RFCBT shares a range of similarities to other psychological interventions such as Behavioral Activation intervention (BA), Mindfulness-Based Stress Reduction (MBSR), and Acceptance and Commitment Therapy (ACT). For example, RFCBT, BA, and ACT all emphasize functional contextual approaches to behaviors, stressing the critical role of avoidance in psychopathology (Umegaki et al., 2022). Whereas, RFCBT particularly aims to eliminate depressive rumination, training individuals to self-monitor their habitual rumination and enabling them to build new adaptive habits (Feldhaus et al., 2020). As a result, it remains unclear to what extent the RFCBT overlaps with other psychological interventions, and which elements of RFCBT are especially effective in addressing depressive symptoms. Future studies could explore dismantling these therapies to identify their effective components or to conduct direct comparisons among these components.

Secondly, it is uncertain which group of people could benefit most from the RFCBT. According to Langenecker et al. (2024), quantities of youths did not exhibit a significant decrease in rumination tendencies. Clinical observation suggests that the youths with limited awareness of their rumination cues and processes found it challenging to adhere to the RFCBT. Therefore, the RFCBT might be less effective and suitable for individuals with limited metacognitive skills. Additionally, Watkins and Nolen-Hoeksema (2014) suggested that the environment played a critical role in the formation of depressive rumination. Therefore, the environment with high stress and large barriers to prevent goal achievement may affect the treatment efficacy. In Langenecker et al.‘s (2024) study, the majority of youth participants were from middle and upper socioeconomic strata, with few from the lower socioeconomic strata. Consequently, it is not quite clear whether high degrees of familial and environmental stress would affect the treatment effect. Further research could explore which people may not benefit from RFCBT and RFCBT could modified to suit these people’s special needs.

Regarding the neurobiological evidence related to the RFCBT, the sample size of the studies was not large enough and thus limited the statistical power and generalizability of the findings. Additionally, potential confounding factors may affect the study results. Bessette et al. (2020) mentioned that participants in the control group may also receive some form of other psychotherapy interventions. Jacobs et al.‘s (2016) study may be also affected by confounding factors since participants may receive ongoing psychosocial and pharmacological treatment. Likewise, the participants allocated to the RFCBT had higher baseline rumination levels (Langenecker et al., 2024). Furthermore, changes in network connectivity can be interpreted in multiple and potentially contrasting ways, such as the disease modification model or disease compensation model (Bessette et al., 2020; Langenecker et al., 2024), current data might not be able to clearly distinguish between these processes. These limitations may constrain the clear understanding and validation of the neural mechanism of RFCBT, therefore, future studies with larger sample sizes, and more rigorous experimental designs are required to fully explore the underlying mechanisms of the RFCBT.

Finally, considering that rumination is identified as a transdiagnostic process, future studies could explore the therapeutic efficacy of RFCBT across a broader spectrum of mental disorders such as generalized anxiety disorder, post-traumatic stress disorder, and a co-morbidity between depression and anxiety, of which rumination is a key contributing factor, to broaden the scope of applicability of RFCBT.

## 6 Conclusion

This review provided a comprehensive understanding of the treatment effects of RFCBT on depression. 10 out of 12 studies reported that RFCBT could significantly reduce depressive symptoms. Overall, RFCBT might be a promising intervention for managing depression, which deserves future investigation. However, when compared to the active controls such as mindfulness-based interventions (MBI), standard CBT, and psychoeducation, RFCBT did not show substantial superiority in the three studies. This highlights the importance of future studies to compare RFCBT with other psychological interventions. Further process-outcome research is required to explore the mechanisms of change and determine the specific elements within RFCBT that result in treatment effectiveness. In addition, studies also revealed the neurobiological foundation of RFCBT in alleviating depressive symptoms, suggesting that RFCBT cultivates a neuro-environment, which contributes to prolonged remission and enhanced regulatory abilities.
